# Performance of Fuzzy Multi-Criteria Decision Analysis of Emergency System in COVID-19 Pandemic. An Extensive Narrative Review

**DOI:** 10.3390/ijerph18105208

**Published:** 2021-05-14

**Authors:** Vicente Javier Clemente-Suárez, Eduardo Navarro-Jiménez, Pablo Ruisoto, Athanasios A. Dalamitros, Ana Isabel Beltran-Velasco, Alberto Hormeño-Holgado, Carmen Cecilia Laborde-Cárdenas, Jose Francisco Tornero-Aguilera

**Affiliations:** 1 Faculty of Sports Sciences, Universidad Europea de Madrid, Tajo Street, s/n, 28670 Madrid, Spain; josefrancisco.tornero@universidadeuropea.es; 2 Grupo de Investigación en Cultura, Educación y Sociedad, Universidad de la Costa, Barranquilla 080002, Colombia; 3 Studies Centre in Applied Combat (CESCA), 45007 Toledo, Spain; ajhh1983@gmail.com; 4 Grupo de investigacion en Microbiologia y Biotecnologia (IMB), Universidad Libre, Barranquilla 08002, Colombia; eduardoi.navarroj@unilibre.edu.co; 5 Department of Health Sciences, Public University of Navarre, 31006 Pamplona, Spain; pablo.ruisoto@unavarra.es; 6 Laboratory of Evaluation of Human Biological Performance, School of Physical Education and Sport Sciences, Aristotle University of Thessaloniki, 57001 Thessaloniki, Greece; dalammi@phed.auth.gr; 7 Education Department, Universidad Antonio de Nebrija, 28240 Madrid, Spain; abeltranv@nebrija.es; 8 Vicerrectoría De Investigación e Innovación, Universidad Simón Bolívar, Barranquilla 080005, Colombia; cacelaca6@gmail.com

**Keywords:** fuzzy decision analysis, decision making, uncertainty, multi-criteria, emergency, COVID-19

## Abstract

The actual coronavirus disease 2019 (COVID-19) pandemic has led to the limit of emergency systems worldwide, leading to the collapse of health systems, police, first responders, as well as other areas. Various ways of dealing with this world crisis have been proposed from many aspects, with fuzzy multi-criteria decision analysis being a method that can be applied to a wide range of emergency systems and professional groups, aiming to confront several associated issues and challenges. The purpose of this critical review was to discuss the basic principles, present current applications during the first pandemic wave, and propose future implications of this methodology. For this purpose, both primary sources, such as scientific articles, and secondary ones, such as bibliographic indexes, web pages, and databases, were used. The main search engines were PubMed, SciELO, and Google Scholar. The method was a systematic literature review of the available literature regarding the performance of the fuzzy multi-criteria decision analysis of emergency systems in the COVID-19 pandemic. The results of this study highlight the importance of the fuzzy multi-criteria decision analysis method as a beneficial tool for healthcare workers and first responders’ emergency professionals to face this pandemic as well as to manage the created uncertainty and its related risks.

## 1. Introduction

From the end of November 2019 to the present (February 2021), the world is facing one of the most significant pandemics of the last two generations. Every day around the world, thousands of people are dying, and hundreds of thousands are becoming infected with this new coronavirus, characterized by its highly contagious nature [1]. In humans, it usually causes respiratory infections that can range from a common cold to severe illnesses such as the Middle East respiratory syndrome (MERS) or the severe acute respiratory syndrome (SARS) [2]. The recently discovered coronavirus (Sars-CoV-2) has caused the actual pandemic (COVID-19), with a greater ratio of mortality and contagiousness than its predecessors [2]. Unawareness and the unknown nature of the Sars-CoV-2 facilitated its rapid spread, leading to 102 million cases and 2.2 million deaths worldwide. In a matter of short time, the virus has been rapidly spreading from Asia to Europe, North America, the Middle East, Africa, and Latin America. Some countries began preparing their health systems for the increase in severely ill patients and implemented stringent measures to prevent the contagion from spreading, such as closing borders, pre-emptive isolation, and quarantine [2]. Depending on these measures adopted, each country has suffered the appearance of different waves of infection. Globally, three key and common worldwide turning points can be highlighted for these waves. The first is located between May and April 2020, the second between July and August, and a third between December and January [3].

From each of these waves, governments, countries, and societies have been learning and improving their response systems. Yet, given the unpredictability of the virus, its behavior, somatological diversity, the recent appearance of new virus variants as the British [4], we are still suffering many new challenges to the healthcare system worldwide and their sustainable performance. In this line, COVID-19 among its waves has set numerous operational, logistical, organizational, and moral–ethical requirements before management, healthcare workers, and associates. Likewise, and seeing the complications in the processes of the distribution and application of the COVID-19 vaccine, it is more than expected that a fourth wave will occur [5], which, seeing the accumulated total active cases, is more than likely to produce saturation of the healthcare system [6]. Therefore, it is required to improve the organization of specialized training for medical personnel by emerging conditions and the conversion of facilities for patient accommodation. Governments and health organizations should also be concerned about the procurement of medical equipment, information and communication management, as well as continuous monitoring of healthcare systems. The availability of healthcare to specific groups of the population in new conditions should also not be neglected [7]. Thus, given the looming epidemiological spectrum and the shadow it has left, it is strictly necessary to continuously analyze the strengths and weaknesses of the healthcare systems and decision-making processes worldwide, which would lead to an adaptative, efficient model that can give speed, agility flexibility and a prompt response, especially on qualified medical personnel. To confront this crisis situation, decision-making models are currently being used in hospital emergencies by providing essential information regarding the prediction of spreading, the evaluation of different factors, determining the weights of criteria, and re-organizing the healthcare systems [7]. In this sense, fuzzy-based decision models, based on the theory proposed by Zadeh in 1965, have also a significant application in healthcare systems, considered as a more flexible and dynamic approach and—at the same time—a more sensitive procedure to real-life scenarios [8,9].

This paper is structured as follows: After the introduction, in Section 2, the basic principles of MCDA and fuzzy MCDA methods are briefly discussed. Section 3 includes the applications of fuzzy MCDA techniques currently applied in hospital emergencies during the first pandemic wave. Section 4 and Section 5 suggest possible implications in first responders’ emergency professionals, as well as in other areas, while Section 6 presents future implications of this procedure. Finally, the obtained results are shortly presented in Section 7.

### Research Methodology

In this paper, a literature search using primary sources, such as scientific articles, and secondary, such as bibliographic indexes, databases, and web pages, was conducted. More specifically, PubMed, Embase, SciELO, Science Direct Scopus, and Web of Science were used, employing keywords including COVID-19, coronavirus 2019, SARS-CoV-2, 2019-nCoV, fuzzy MCDA, multi-criteria decision analysis, MCDM + COVID-19, fuzzy MCDM + COVID-19. We used articles published from 10 January 2020 until 25 March 2021, although some previous studies were included to analyze the basic principles of the multi-criteria decision analysis method in general. The following exclusion criteria were used: i. studies with old data (out of COVID-19), and ii. present inappropriate topics, being not pertinent to the main focus of the review. The information treatment was performed by the eight authors of the review. Finally, the articles were discussed by the authors to write this review.

## 2. Basic Principles in Fuzzy Multi-Criteria Decision Analysis

Multi-criteria decision analysis (MCDA) is currently one of the most well-known topics of decision making. In a multi-criteria decision-making problem, several alternatives are evaluated with respect to several criteria to select the best feasible alternative(s) [10]. MCDA is an effective tool for helping decision-makers, such as first-response emergency professionals, to find an optimal alternative when solving complex selection problems including decisions that may involve life and death. This technique in particular, MCDA, uses a decision matrix to provide a systematic analytical approach for establishing the criteria and rank of the considered alternatives. Specifically, according to Diaby and Goeree (2014) [11], the fundamental steps in a multi-criteria decision analysis are the following: first, to define the problem and generate alternatives; second, to identify the criteria to compare alternatives; third, to gather value judgments on the relative importance of the criteria; forth, to screen/eliminate clearly inferior alternatives; fifth, to determine the performance of the alternatives for the criteria; and sixth, to rank/select the final alternative(s).

MCDA would refer to a structured decision-making process that allows assessing and easily synthesizing available evidence to facilitate the decision process and estimate risks involve. In other words, MCDA would refer to any decision-making technique involving the numerical analysis of alternatives by determining the relevant criteria and alternatives; assessing the relative impact of the alternatives on those criteria; and finally ranking the alternatives [10]. The initial framework, introduced by Saaty [12], (i.e., the analytical hierarchy process—AHP) used pair-wise comparison matrices and provided weights for the different criteria of decision making. More recently, Rezae developed the best–worst method [13,14]. In this case, the best against the other criteria—and the other against the worst criterion—are evaluated using linear and non-linear models, presenting the advantage of including a lower number of comparisons [15]. Based on the best–worst method, more recent approaches are also proposed [16].

In hospital settings, MCDA models (e.g., AHP, and the decision-making trial and evaluation laboratory method—DEMATEL) have previously been used towards a better preparation for major disasters, by calculating and describing the interrelations of criteria and sub-criteria weights [17]. Additionally, the technique for order of preference by similarity to ideal solution (TOPSIS) was used to evaluate healthcare facilities in the study of Hosseini et al. [14]. More recently, a hybrid model—the A.D.T. model—based on the three above-mentioned ones was proposed to evaluate the disaster readiness of the emergency departments for disaster situations, facilitating the consideration of how well the health system or program is performing in Colombia [18]. Similar “conventional” MCDA methods were also applied in hospitals during this pandemic, which aimed to map areas susceptible to infection [19] as well as to analyze the risk of infection in different urban areas [20].

However, real-world problems are usually too complex and often include imprecision or vagueness, and are inherent to data or information, thus, requiring a more “sensitive” approach. In this context, if MCDA refers to making decisions in the presence of multiple and usually conflicting criteria, fuzzy decision-making analysis is precisely used where vague and incomplete data exist for the solution [21]. Indeed, fuzzy multi-criteria decision-making (fCMDM) analysis is one of the most popular problems handled by researchers in the literature [22]. The application of fuzzy sets has been widely used in several scientific areas, such as computer science, artificial intelligence, social sciences, management, etc., as an extension of the conventional MCDA methods previously mentioned [23].

Similar to MCDA, fMCDM assesses alternatives with respect to the criteria through either a single decision maker or a committee of decision makers, where suitability of the alternatives is based on predetermined criteria, and the importance or weights of such criteria [9,10,24,25,26]. However, the distinctive feature of fMCDM is that a fuzzy number does not refer to one single value but rather to a connected set of possible values, where each possible value has its weight between zero and one [10]. In other words, this can be understood as an extension of the Boolean logic, based on true—1 and false—0 values, with nothing in between. This is important because fuzzy numbers allow to consider uncertainty by using intervals. In other words, a fuzzy number equals a fuzzy interval [21].

In sum, fMCDM conveys uncertainty and risk that may compromise the decision-making process. In this context, the fuzzy logic provides a useful way to approach decision making [24]. Furthermore, most decision making in daily life would fall within this category in including emergency responses in COVID-19 [26]. Finally, in a relatively recent study, Kahraman et al. (2015) [24] surveyed the latest status of fuzzy multi-criteria decision-making methods and found the following two main methods: fuzzy multi-attribute decision-making (fMADM) and fuzzy multi-objective decision-making (fMODM). However, most of the publications were on fMADM since there are plenty of classical multi-attribute decision-making methods in the literature, particularly in the context of first-responder emergency professionals.

## 3. Applications of Different Fuzzy Techniques on First COVID-19 Wave in Hospital Emergencies

The prevention and control of COVID-19 has become a global priority throughout the world. The measures adopted by the different governments in terms of containment and control continuously may affect the mental and general health as well as the living conditions of the citizens [1,2]. A gradual adaptation process scientifically agreed to achieve a return and adaptation to the “new normal” [19].

Artificial intelligence, big data as well as mathematical modeling and processing, have helped enormously to justify and understand the behavior of the virus from an epidemiological point of view. Indeed, providing important information on various parameters and their effects in different scenarios. Yet, these traditional models and mathematical analyses are insufficient in the case of infections caused by different strains of the virus [27].

To solve this new difficulty, integrative models such as “fuzzy logic” may be a useful mathematical tool for handling various types of uncertainties as in the case of these new virus strains. The fuzzy logic framework can be used in different types of disease diagnosis, mainly chronic non-communicable, but few infections in which the knowledge of the doctors and experts is represented on behalf of the symptoms and diseases [28]. In addition, fuzzy logic has been used for the increase, optimization, and preparation of hospitals placed in low- and medium-resource countries facing natural disasters, or as in the present case, with COVID-19 [18].

Under the COVID-19 umbrella and the fuzzy logic framework, works such as those of Shaban et al. [29] can be made an example of. A patient detection model based on laboratory techniques (RT-PCR) and radiological diagnostic tests using a fuzzy inference engine, and a deep neural network technique, was proposed. The experimental results have shown that the proposed hybrid diagnostic strategy (HDS) outperformed the other competitors in terms of the average value of precision, recovery, and F-value in which it provides approximately 97.658%, 96.756%, 96.55%, and 96.615%, respectively, with the lowest error value of 2.342% [30,31]. Furthermore, Sethy and Behera [32] used the CNN model to classify two classes that achieved an accuracy rate of 95.38%. Xu et al. [33] used two CNN three-dimensional classification models for two classes of chest images, obtaining an 86.7% precision with the data collected from the two classes. Wang et al. [34] analyzed 325 COVID-19 CT images and 325 CT images obtaining an 89.5% success rate using the inception transfer-learning model.

From a pharmacological perspective, studies such as those of Yildirim et al. [8] used Diffuse PROMETHEE and VIKOR fuzzy systems to evaluate the most suitable pharmacological interventions for COVID-19 treatment. Favipiravir (FPV), lopinavir/ritonavir, hydroxychloroquine, interleukin-1 blocker, intravenous immunoglobulin (IVIG), and plasma exchange were analyzed. The results showed that plasma exchange was the most preferred alternative, followed by FPV and IVIG, while hydroxychloroquine was the least favorable. The authors conclude that either PROMETHEE or VIKOR fuzzy systems are optimal tools to help decision makers choose the appropriate treatment technique for the management of COVID-19.

In this line, the work of Batur Sir and Sir (2021) [35] was based on using a multi-criteria decision-making methodology, aiming to guide physicians in the selection and classification of various alternatives for the treatment of pain in patients with COVID-19. First, the criteria and sub-criteria that affect clinical treatment preferences were defined. Then, the weight values were determined for these criteria, since they have different degrees of importance for the problem. At this stage, hesitant fuzzy linguistic term sets (HFLTSs) are used and therefore experts can convey their ideas more accurately. In this first phase of the study, an integrated HFLTS and analytic hierarchy process (AHP) method were used. Subsequently, possible treatment alternatives were evaluated using the vise kriterijumska optimizacija I kompromisno resenje (VIKOR) method. Based on the results obtained when considering expert evaluations, the most preferred treatment is the administration of paracetamol, followed by interventional treatments, opioids, and non-steroidal anti-inflammatory drugs (NSAIDs).

From the point of view of prevention, control, and personal safety equipment regarding COVID-19, Fu et al. [36] used fuzzy logic programming to identify and summarize the set of common medical products used in hallways and household disinfectant products based on recommendations from the World Health Organization and the Center for Disease Control (CDC). Among them, thermometers, disinfectants, face masks, gloves, or oximeters. Yet, these results helped to guide the development and programming of disinfectant products and sanitize protocols for public and private places.

In the primary sector, Palou et al. [37] used the fuzzy delphi method (FDM) and the fuzzy analytical hierarchy process (FAHP) to study the impact of the COVID-19 pandemic on the Iranian poultry supply chain. The authors identified disruptions associated with each stage of the production chain, suggesting that the pandemic has further affected input supply as a stage in the poultry supply chain. This is probably because the poultry industry is highly dependent on the flow of inputs. Furthermore, the governance of the supply chain was seriously affected due to the persistence of the pandemic.

In hospital emergencies, distributive justice and the maximization of global benefit among patients is a decision based on triage. As seen in the previous waves of COVID-19, an overwhelm of the outpatient facilities, emergency departments, hospitals, and intensive medical services has been seen [38]. It impacts available resources, both at the level of structures, equipment and professionals, with serious consequences on the results of patients, their families, their health professionals, and society in general. There is, temporarily, an imbalance between clinical needs and effective availability of health resources.

This exceptional situation should be handled like “medicine of catastrophe” [39], applying exceptional crisis care based on distributive justice and in the adequate allocation of health resources. Indeed, healthcare systems and providers must be prepared to obtain the greatest benefit of limited resources and mitigating harm to people, to the healthcare system, and society.

In the case of the current pandemic, hospitals are the cornerstone, and emergency care, which is essential to safeguard the lives of millions of patients. However, both hospital resources and emergency room beds are limited. Thus, some organizations such as the SMYCIUC, formed by specialists in intensive medicine, have made some guidelines for the objectification of the decision-making process of the patient in hospital emergencies [40], they are as follows:

- Ensure the presence of an emergency contingency plan that plans the distribution of patients between geographic areas;
- Ensure the possibility of transfer or referral to another hospital with available and greater resources;
- If the patient cannot be transferred, assess the possibility of expanding the capacity of local emergency hospitals (E.g., Zendal Hospital in Madrid, Spain, recently opened due to the 9% weekly exponential number of cases) [41];
- In the case of disproportion between demand and possibilities, it is lawful to establish an admission triage among patients, based on the principle of distributive justice, avoiding following the usual criterion of “the first in arriving is the first to receive assistance”. These triage protocols consist of a system of rules that are applied in the framework of scarce resource situations to help make decisions fairly and transparently [40,42,43] (see Table 1);
- Solidarity between centers is a priority to maximize the common good for above the individual good.

## 4. Fuzzy Multi-Criteria Decision Analysis in First Responders’ Emergency Professionals

Considering the COVID-19 pandemic as a “natural disaster”, first responders’ emergency professionals are on the frontlines of supporting communities. Along with medical staff and paramedic teams previously analyzed, mental health counselors, police and law enforcement officers, as well as military personnel were the first to confront the challenges of the ongoing coronavirus outbreak [44]. Although a considerable number of studies have been devoted to understanding the mental health consequences of this period on the above-mentioned professional groups [45,46], less attention has been given to the decision-making process in threatening situations that may depend on a variety of factors, such as the governments’ targeting, cultural influences, colleagues’ opinions, and subjective judgments.

**Figure 1:**
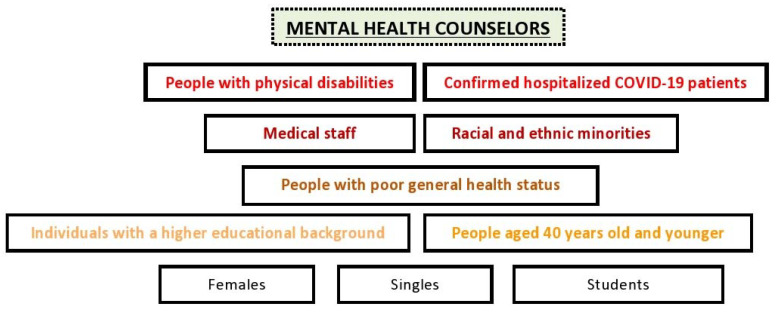
Priority groups for mental health counselors during the COVID-19 pandemic.

This pandemic is a unique threat to mental health in the general population [47], while people with physical disabilities and hospitalized patients with confirmed COVID-19 are considered more vulnerable [48,49]. The exacerbation of pre-existing mental issues, although only significant in patients with mild symptoms, is also obvious [43]. In this situation, mental health counselors have to deal with a wide range of psychiatry issues, including anxiety and depression [50], in both non-infected and COVID-19 survivors. Specific population groups such as females, people aged 40 years old and younger, singles, students, people with poor general health status, racial and ethnic minority groups, individuals with a higher educational background, and medical staff, were most associated with depression symptoms and stress-related disorders [47,51,52] (Figure 1). Moreover, job loss or income insecurity has negatively affected mental health in adults [53].

Considering the above-mentioned observations, the establishment of different weights of criteria to identify the priority groups, as applied by the fMCDM method, towards the implication of intervention strategies for moderating the negative impact of COVID-19 on mental health may have significant importance. Complementarily, individuals at a higher risk of suicide should be identified as part of a diagnostic approach and get additional support. Nevertheless, to the author’s knowledge there are no available data referring to the fuzzy method applied by mental health workers during the first or the second pandemic wave while, in general, the respective approaches in social psychology research are scarce [54]. Since the long-term adverse consequences of the coronavirus outbreak on mental health are not yet fully realized, while historically it may last longer compared to the physical health effects, the application of the fMCDM method should be also extended to this emergency group in terms of a comprehensive response to this pandemic.

The duties and responsibilities of police and law enforcement officers consist of fighting the crime and enforcing the law. This unprecedented situation characterized by uncertainty and disorientation created additional duties, namely, to protect communities and prevent the spread of COVID-19. These actions include the encouragement of social distancing and use of face masks, enforcing lockdown rules, imposing travel restrictions, maintaining public order and crowd control, and, additionally, ensuring the safety of health professionals and equipment. One of the main challenges of this crisis for this professional group, however, is to maintain communication with the citizens, especially those affected, create a connection with essential social services for the more vulnerable individuals, provide humanitarian support, while also reducing the rates of specific types of crimes (e.g., domestic violence) [55,56].

In this sense, policing strategies should probably be reconsidered to prioritize responsibilities [57]. In fact, a previous study recommended changes in policy styling during various stages of disasters, aiming to maximize effectiveness [58]. In a study that applied the “least–worst” multi-criteria decision-making method (i.e., actions of high risk with possible negative consequences), it was found that police officers’ gender and previous military experience influenced this process [59]. In the lack of studies focusing on the fMCDM method during the pandemic, characterized by time pressure, incomplete knowledge and limited resources by police and law enforcement officers, individual differences such as personality traits, intuitive styles, and sacred values should also be taken into account.

The MCDA method has also profound implications in the military decision process as the problems faced by the personnel are complex and particularly risky, frequently involving serious issues, for instance, the country’s defense. Selecting the bases site, weapon systems, training equipment, or even the location of military hospitals, and organizing peacekeeping missions have previously used the MCDA method, often with fuzzy logic [60,61,62]. Being highly skilled in disaster management, military personnel have a significant contribution in tackling the COVID-19 pandemic. The transportation of medical supplies and health workers, disinfection of public places, deploying military medical professionals to hospitals, producing medical supplies, or even testing the effectiveness of possible vaccines, among others, are some of the contributions to the frontline services. Since no data regarding the application of the fMCDM method in military professionals during this emergency period are available, future possibilities may include the advisory and the more active role based on previous experience in crisis leadership [63].

## 5. Application of Fuzzy Multi-Criteria Decision Analysis in Other Areas

Fuzzy multi-criteria decision systems have been used since the early 1990s. They were firstly used in the commercial–economic sphere to assess and analyze the liquidity and debt capacity of people applying for a credit card in Germany. Ten years after this first attempt, MCDM was applied including some modifications to assess military weapon systems [64]. Yet problems arrived with it, the first of these, which was solved with the traditional MCDM, was the multiple objectives of the evaluations of these weapons systems; the second problem referred to the representations of the systems, which meant the inclusion of fuzzy MCDM techniques. With the use of triangular fuzzy number scales, a judgment matrix was elaborated to be able to carry out similes and verifications. Thus, it was possible to achieve an optimal index to obtain the highest level of satisfaction from the person who had to make the decision [9].

Indeed, MCDM has also been used in the selection of suppliers through the application of the fuzzy DEMATEL, which is based on the study and development of matrices and diagrams until reaching a structure of causal relationships. This technique allows evaluating a series of criteria that are compared in pairs and, later, classified into levels. Thus, allowing to establish direct relationships between these criteria to obtain the most appropriate in each case, including qualitative and quantitative aspects, to choose the more positive [65].

In other areas such as knowledge management, it is known that different variables may impact MCDM success. Yet, with more traditional prediction systems there is a tendency to forget qualitative nature variables as factors inherent to the people and the environment [60]. With the fuzzy multi-criteria decision approach, these limitations have been overcome and it has been possible for organizations to identify all the options and variables that will determine the success or failure of the implementation of knowledge management. These are adapted to either the people that make up the institution as well as the organization itself, by analyzing the critical and essential factors within a finite set of alternatives, which are classified and valued using techniques such as simple additive weighting (SAW) or the analytical hierarchy process (AHP), among others [66].

Furthermore, other contexts in which the fuzzy multi-criteria decision method system has been used is in those related to energies, either renewable or traditional. Energy industries have intended to offer a cogeneration model that integrates different energies for the consumer in an efficient way [67]. Yet, the traditional single-criterion analysis model has been rejected once it has been analyzed that decision-making to achieve a trigeneration system, that is, the generation of electrical or mechanical energy simultaneously with heat or cooling with the source of heat only, has more than one feasibility study variable, which was usually economic [68]. Since the implementation of the MCDM model for the study and analysis of the decision criteria, it has been possible in this field to make a selection of weaknesses that appear first. Thus, giving way to the acceptable alternatives and their subsequent classification and analysis, and offering the most valuable alternative is essential. In this line, the ordering of preferences by similarity with the ideal solution (TOPSIS) or the weighted sum method (WSM) are the methods of recent use in this field [69].

## 6. Future of the Fuzzy Multi-Criteria Decision Analysis

Although a relatively recent research method, the use of multi-criteria methodologies is still emerging and exponentially becoming widespread in the international scientific community [54] to approach difficult decision-making situations in a simpler way. As shown in previous studies, it has been applied successfully as an optimal decision-maker methodology to address complex situations [68,70]. Not only in engineering has commercial or energy fielded, but also to become relevant in the medical sector. The reviewed applications of fMCDA and fMCDM reaffirm their huge contribution in real-life scenarios such as the COVID-19 pandemic. The future in fMCDA must become clear, and the more we understand the importance of fMCDA decision sciences within the different scientific fields, the earlier we will succeed in the large variety of fuzzy sets [9], obtaining an improvement in social health of population [1]. Actually, this is an important fact to consider, specially in emergency first interventions team as practitioners, nurse or psychologies [71,72].

Uncertainty is one of the biggest challenges in different areas. Most networks and especially new settings such as those created in the medical field, for example, the present outbreak of a new virus, are affected by various sources of uncertainty. This uncertainty becomes hard to handle when it leads to increased variability of the effects on patients. Therefore, making explicit the risk linked to the future decision action plan may be an asset for supporting the decision of the health managers. The research on input uncertainty can be added to the study of COVID-19. Certain methods, such as TOPSIS, can manage this situation and several decision-making problems. Also, these new methods were applied in fuzzy environment and could overcome the drawbacks of the existing methods for maldistributed decision making.

As it was mentioned above, fMCDA has been used to reduce uncertainty and shortcomings of the decision-making progress [73]. Regarding its future, fMCDA could avail to deal with dubious and subjective data, minimizing ambiguity and dubiousness during the evaluation process in future matters such as the present pandemic. In this line, the hesitant fuzzy set theory coupled with the TOPSIS tool was applied to finding the most significant risk factor for the spread of the COVID-19 disease, and again TOPSIS to compare the confusion about choosing an effective diagnostic method in order to compare the SARS-CoV-2 diagnostic tests with each other, or evaluation of its risk assessment [74].

As we continue to understand the behavior and characteristics of COVID-19, future research directions will be diverse. In this line, the application to the psychology area, highly impacted in the COVID-19 pandemic [75,76]. In the medical sector, decision-makers have to evaluate both the qualitative and quantitative criteria. Detecting infected cases and tracking people who may be infected is a daunting task, in which technology may play an effective role.

Future fuzzy multi-criteria decision analysis methodology could be used to explore different variables, such as isolation planning, the location for quarantine centers, safe nursing homes, safe homes, safe mask, and an epidemic controlling model and intensive care unit beds augmentation model for COVID-19 hospitals to assure care for a large number of people. Research involving different lockdown models or controlling the population who has developed antibodies of COVID-19 can guarantee better distribution of personnel for COVID-19 management. Detailed research and data analytics will help in understanding the level of community spread. Moreover, new variants of the SARS-CoV-2 virus are detected every week, so the decision criteria must ensure that the current testing, treatment, and vaccines are still effective or examine new ways of tackling the virus effect. The use of the fuzzy-based MCDM method can also be adjusted according to the specific situation of the patient.

## 7. Conclusions

The present systematic review examined the existing and possible applications of the fuzzy MCDA method for facing issues and challenges related to the ongoing COVID-19 pandemic, as realized by healthcare workers and various professional groups characterized as first responders, as well as in other areas. Additionally, specific results of the application of this method during the first pandemic wave were discussed. Finally, further suggestions related to this methodology to confront the uncertainty caused by this crisis were presented.

## Figures and Tables

**Table 1:** Decision based on triage for hospital emergencies.

Triage Patient Categorization	Patient Description	Recommendation
Patient with priority I	Critical and unstable patients. Need of intensive monitoring and treatment which cannot be provided outside the ICU (invasive mechanical ventilation, continuous renal clearance …).	First priority for admission intensive care.
Patient with priority II	Require intensive monitoring and may require interventions immediately. They are patients who will not be ventilated invasively but with high oxygen therapy requirements with PaO2/FiO2 less than 200 or less than 300 with failure of another organ.	After patients with priority 1. Encourage admission in intensive care.
Patient with priority III	These are unstable and critical patients who have little chance of recovery from their underlying or acute illness. They can receive intensive treatment to alleviate their acute illness, but also establish their therapeutic limits, such as not intubating and/or not attempting resuscitation.	In cases of crisis, will not be admitted to units of intensive care.
Patient with priority IV	Patients whose admission is not generally indicated due to a minimum benefit or unlikely due to low-risk disease. Patients whose terminal illness and irreversible makes his death imminent.	In cases of crisis, will not be admitted to units of intensive care.

## Data Availability

Not applicable.
